# Association of the Maternal Folic Acid Supplementation with the Autism Spectrum Disorder: A Systematic Review

**DOI:** 10.1055/s-0041-1736298

**Published:** 2021-11-16

**Authors:** Adalberto Cruz Sampaio, Francisco Facundo de Matos, Lucas de Lucena Lopes, Ítalo Marcelo Maia Marques, Ravel Moreira Tavares, Marcus Vinicius de Macedo Fernandes, Maria Raquel Vieira da Silva Teixeira, Alessandra Bezerra de Brito, Andrea Couto Feitosa, Tarciana Oliveira Guedes, Magaly Lima Mota

**Affiliations:** 1 Department of Medicine, Faculdade de Medicina Estácio de Juazeiro do Norte, Barbalha, CE, Brazil; 2 Department of Nursing, Faculdade Integrada de Araguatins, Juazeiro do Norte, CE, Brazil; 3 Department of Nursing, Centro Universitário Dr. Leão Sampaio, Crato, CE, Brazil; 4 Department of Nursing, Centro Universitário Dr. Leão Sampaio, Juazeiro do Norte, CE, Brazil

**Keywords:** autistic spectrum disorder, folic acid, pregnancy, supplementation, transtorno do espectro autista, ácido fólico, gravidez, suplementação

## Abstract

**Objective** To analyze the scientific production regarding maternal folic acid (FA) supplementation and its relationship with autistic spectrum disorder (ASD).

**Data Sources** We performed unrestricted electronic searches in the BIREME virtual bank, Virtual Health Library (VHL) and Medical Literature Analysis and Retrieval System Online (MEDLINE/PubMed) databases.

**Selection of Studies** For sample selection, articles that met the proposed objectives were included, published in English, Spanish and Portuguese, the use of Health Sciences Descriptors (DeCS): *autistic* OR *autism* AND *autism spectrum disorder* AND *folic acid* , AND, with the use of the Medical Subject Headings (MeSH): autistic OR *autism* AND *autistic spectrum disorder* AND *folic acid* .

**Data Collection** Data extraction was performed by the reviewers with a preestablished data collection formulary.

**Data Synthesis** The Preferred Reporting Items for Systematic Review and Meta-Analysis Protocols (PRISMA-P) was used based on a checklist with 27 items and a 4-step flowchart.

**Results** A total of 384 articles was found by the search strategies, of which 17 were eligible following the pre-established criteria. The main findings of the present review point to maternal FA supplementation in the pre-conception period and beginning of pregnancy as a protective effect in relation to ASD, which should be indicated in this period as prevention to the problem.

**Conclusion** According to the research analyzed, more studies are necessary to know its effects on pregnancy, since the consumption of excessive FA may not be innocuous.

## Resumo

**Objetivo** Analisar a produção científica a respeito da suplementação de ácido fólico (AF) materno e sua relação com o transtorno do espectro autista (TEA).

**Fontes de Dados** Realizamos buscas eletrônicas irrestritas nas bases de dados do banco virtual BIREME, Biblioteca Virtual em Saúde (VHL) e Medical Literature Analysis and Retrieval System Online (MEDLINE / PubMed).

**Seleção dos Estudos** Incluímos os artigos publicados em inglês, espanhol e português, com o uso dos DeCS: *autistic* OR *autism* AND *autism spectrum disorder* AND *folic acid* , e com o uso dos Medical Subject Headings (MeSH, na sigla em inglês): autistic OR autism AND Autistic Spectrum Disorder AND folic acid “.

**Coleta de Dados** A extração de dados foi realizada pelos revisores com um formulário de coleta de dados pré-estabelecido.

**Síntese dos Dados** Foram usados os itens de relatório preferidos para protocolos de revisão sistemática e meta-análise (PRISMA-P) com base em uma lista de verificação com 27 itens e um fluxograma de 4 etapas.

**Resultados** Foram encontrados 384 artigos pelas estratégias de busca, dos quais 17 eram elegíveis segundo os critérios pré-estabelecidos. Os principais achados da presente revisão apontam para a suplementação de AF materno no período de preconcepção e início da gravidez como efeito protetor em relação ao TEA, que deve ser indicada neste período como prevenção do problema.

**Conclusão** De acordo com as pesquisas analisadas, mais estudos são necessários para conhecer seus efeitos sobre a gravidez, uma vez que o consumo excessivo de AF pode não ser inócuo.

## Introduction

Autism Spectrum Disorder (ASD) is characterized by persistent deficits in social interaction and communication, with the presence of repetitive interests and activities. Considered as a neurodevelopmental disorder, it can manifest with extremely variable phenotypes, from severely compromised individuals to independent individuals. 1 2

The World Health Organization estimates that 1 in 160 children has ASD. The use of preconception folic acid (FA) should be indicated at least 2 months before conception and in the 1 ^st^ 2 months of pregnancy, as it has a protective effect against open defects of the neural tube. 3

Since 2004, in Brazil, the Ministry of Health, through the National Health Surveillance Agency (ANVISA, in the Portuguese acronym), adopted the Collegiate Directorate Resolution (RDC, in the Portuguese acronym) No. 344 of December 13, 2002, establishing mandatory FA fortification in wheat flour and corn to reduce the prevalence of maternal anemia and defects of the neural tube. This fortification of synthetic FA may have generated a population group with high serum levels of nonmetabolized FA. This finding occurs when > 200mg/day is ingested. 4

The theme has great importance in the social context because it involves the patient, their family, the state, and the multiprofessional action, which makes its study fundamental to guarantee the practice of evidence-based health care. The objective of the present review is to describe the relationship between maternal FA supplementation and ASD, according to scientific publications.

## Methods

This is a qualitative exploratory study, of the metasynthesis type. The search for data was performed between February 2018 and February 2020, based on the BIREME virtual bank, the Virtual Health Library (VHL), and the Medical Literature Analysis and Retrieval System Online (MEDLINE/PubMed) databases.

The inclusion criteria were: original articles, systematic reviews, available in full, free of charge, studies with human beings, published in English, Spanish, and Portuguese, which addressed the use of FA related to the occurrence of ASD.

The exclusion criteria used in this study start with free availability, that is, studies published on non-free platforms were excluded from this research. The year of publication was also one of the criteria used in this review, these were strictly maintained between the years 2013 to 2020, another criterion used was the thematic, to exclude all works in which their title and later their summary dealt with a different theme of the association between maternal FA supplementation and ASD.

Studies that depict ASD associated with other drugs or other factors that do not correspond to consumption of supplemental FA were excluded from the study, or those that deal with the consumption of FA without associating it with ASD. The period in which FA is used was also one of the criteria used in the present review, and all those dealing with the consumption of FA at different periods of the periconceptional and gestational period were excluded. Studies with animals and those that portrayed other subjects outside the area of interest of the present research were excluded.

The operationalization of the search for data collection was performed in the BIREME database using Health Science Descriptors (DeCS) linked to Boolean operators and using quotation marks in compound words, ( *autistic* OR *autism* ) AND (“ *autism spectrum disorder* ”) AND (“ *folic acid* ”). In the PubMed database, the search was performed in English using Medical Subject Headings (MeSH) linked to Boolean operators and quotes in compound words, ( *autistic* OR *autism* ) AND (“ *autistic* *spectrum* *disorder* ”) AND (“ *folic acid* ”).

**Fig. 1:**
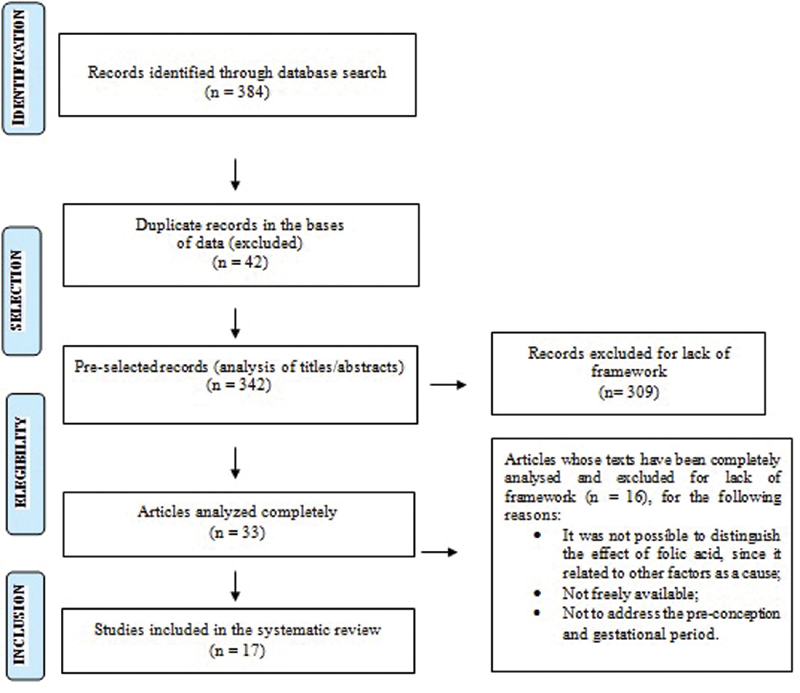
Flowchart of eligible items. Source: Flowchart prepared according to PRISMA recommendations.

The Preferred Reporting Items for Systematic Review and Meta-Analysis Protocols (PRISMA-P) was used based on a checklist with 27 items and a 4-step flowchart. 5 In the present review, the acronym PICO (Patients; Intervention; Comparison; Outcome) was also used to construct the guiding question of the study and to perform the bibliographic search. For the presentation of eligibility criteria, the PRISMA and the table containing information such as authors, year of publication, place and results were used ( Fig. 1 ).

## Results

According to the search strategy adopted, 220 articles were found at BIREME and 164 at PubMed, totaling 384 selected articles, as seen in Fig. 1 , and the studies are presented in tabular form, as seen in Table 1 .

**Table 1:** Characteristics of the articles that composed the body of analysis of the study according to authors, year, place and main results

Authors	Year	Place	Main results
Steenweg-de-Graaff et al. 6	2015	Rotterdam, Netherlands.	The concentration of folic acid at the beginning of pregnancy does not show a direct relationship with autism in the offspring. However, it shows a decrease in traces of childhood autism, related to early folic acid supplementation.
Neggers 14	2014	Alabama, USA.	Autism may be related to the potential association between DNA methylation by high folic acid intake, food fortification with folic acid, and it may also be pointed out as a protective factor for autism. Therefore, the cause of autism could not be affirmed, only possible relations with the problem, being necessary further studies in this respect.
Braun et al. 7	2014	Cincinnati, Ohio, USA	Among the mother-son pairs who used multivitamins with folic acid in the 2 ^nd^ trimester of pregnancy, it was shown to have a beneficial effect in reducing autistic characteristics. When the concentrations of folate in maternal whole blood alone were seen, a protective relationship against the disorder was not observed.
Berry et al. 15	2013	Atlanta, Georgia, USA.	The increasing number of ASD cases, together with the fortification of food folic acid, leads to some aspects of ASD. It may be protective in relation to folic acid supplementation and ASD or it may have an adverse effect of this excessive supplementation, increasing the risk for the problem. As its role is not yet clear regarding its relationship with ASD, future studies are needed.
Schmidt 8	2013	California, USA.	The prevalence of ASD decreases as preconception folic acid is used and in early pregnancy. A 40% decrease in the risk of developing the disorder is observed.
Gao et al. ^21^	2016	Columbia, USA.	Although studies have been observed that indicate a relationship between folic acid supplementation and autism, most of the articles studied in this review suggest that folic acid supplementation in the pregnancy has a protective effect against ASD.
Raghavan et al. 18	2018	Boston, USA.	Based on folate measured in the maternal blood plasma, there is a relationship between increased and decreased folic acid consumption with an increased risk of ASD. However, moderate intake of multivitamins indicates a decreased risk for ASD.
Strøm et al. ^22^	2018	Denmark	The supplementary consumption of preconception folic acid and at the beginning of pregnancy did not present ASD as an outcome. A detailed examination of genetic factors and biomarkers of exposure is necessary.
Wang et al. 9	2017	China	Maternal folic acid supplementation demonstrates protective action against ASD. The consumption of folic acid could allow a significant reduction in the risk of the disorder when compared with those who did not perform the supplementation.
Rangel-Rivera et al. 16	2015	Colombia	Folic acid remains a major ally against structural defects of the central nervous system. There are aspects that affirm its protective effect in relation to ASD, but this study is inconclusive to affirm its protection.
Wiens et al. 13	2017	Iowa, USA.	Excessive consumption of folic acid proves not to be innocuous, high levels of nonmetabolized folic acid denote a negative effect on neurological development, being consequently related to disorders such as ASD.
Moussa et al. 10	2016	Houston, Texas, USA.	Preconception and early pregnancy folic acid consumption show a positive effect in reducing the risk of ASD, but its effects are not known if used during the entire pregnancy.
Neggers 17	2014	Alabama, USA.	Fortification of folic acid associated with preconception folic acid supplementation has shown a close relationship with the increase in the number of ASD cases. Further studies in this area are needed to reach a conclusion.
DeVilbiss et al. 19	2015	Philadephia, USA.	Folic acid consumption has shown a relationship with ASD, and this relationship is both with risk, related to high consumption, and with insufficient intake, given its necessity in neurodevelopment. The dosage of folic acid, vitamin B12 and homocysteine is necessary for a more complete and conclusive evaluation of this relationship.
Surén et al. 11	2013	Norway	Maternal folic acid supplementation between the 4 ^th^ week and before the 8 ^th^ week after the beginning of pregnancy is associated with a lower risk of ASD. However, the inverse association observed represents a causal relationship, indicating that folate deficiency around conception and early pregnancy, or reduced capacity to use available folate, are important causes of ASD.
Tan et al. 12	2019	China	The study demonstrates that maternal use of folic acid and/or micronutrients during pregnancy offer reduced the risk of autistic characteristics in the offspring. Observing characteristic signs regarding social cognition, media, autism behavioral mannerisms, adaptive behavior, coarse motor behavior and problems in gastrointestinal tract of children with ASD. There is also a relationship with the consumption of the micronutrient in pregnancy and vitamin status in children with ASD.
Altamimi 20	2018	Palestine	Through a systematic review, the relationship of ASD with maternal use of micronutrients was evaluated, and it was observed that folic acid can be related to ASD due to its relationship with glutathione, a product of the methionine cycle that depends on folic acid and B12. This element is involved in neuroprotection against oxidative stress and neuroinflammation in the brain. According to studies, glutathione is deficient in children with autism compared with typically developed children.

Abbreviation: ASD, autism spectrum disorder.

Source: Own elaboration, 2020.

The main result of the present review is that the use of FA in the preconception period and in the beginning of pregnancy is effective in preventing ASD. Therefore, early supplementation with FA is beneficial for neurodevelopment and, consequently, acts on the prevention of ASD. The consumption of additional FA at an early stage should be indicated, because in addition to preventing ∼ 70% of neural tube defects cases, it is associated with a protective effect in relation to ASD.

It was also found that excess FA consumption and the gestational period in which it is used can have a negative influence on the occurrence of ASD. Too much intake can be observed, since FA, in addition to being consumed in the usual diet, can also be added in the form of oral supplementation, which, associated with the extended time of use, demonstrates a causative effect for the disorder.

Many studies addressed in the present review bring inconclusive results regarding the effect of FA in relation to ASD, indicating the need for future investigations of maternal FA serum levels and also a detailed investigation regarding its consumption from the 2 ^nd^ trimester of pregnancy, a period considered a risk for its use, in relation to the disorder. All this is relevant, so that optimal doses are indicated according to individual needs and maternal nutritional deficiency.

Of the 15 studies evaluated, 8 were unable to safely determine the effects caused by FA. The majority found that there is an effect on neurodevelopment and even observed a relationship with ASD, but in a contradictory way, which may be protective or even cause the problem. They also point out the need for further studies and that an evaluation of FA in the blood plasma of the mother should be performed to analyze its possible indication, in optimal dose and time.

## Discussion

After the analysis of the eligible articles and following the already defined method, three thematic categories emerged: FA as a protection to ASD, increased risk of ASD in relation to excessive use of FA, and inconclusive association between FA consumption and ASD.

### Folic Acid as Protection against Autism Spectrum Disorder

Studies carried with children diagnosed with ASD whose mothers used FA in the 6 weeks before and after conception pointed to the protective effect of preconception FA and the occurrence of ASD. 6 7 8 Folic acid consumption is related to a decrease in the occurrence of ASD when compared with other mothers who did not have the supplementation. 9 10 Corroborating in the study, the nonuse or deficiency in the folate metabolism in this same period can be pointed out as a cause for the development of autism disorder. 11

A study was conducted in a Chinese population with a total of 416 children with ASD and 201 children with typical development (TD). It concluded that children born to mothers without FA and micronutrient supplementation during pregnancy exhibited more severe social cognitive impairment, such as social communication, behavioral mannerisms, delays in developing raw and adaptive motor behavior, and gastrointestinal problems than children born to mothers who used FA and micronutrient supplements, demonstrating the need for micronutrient supplementation during pregnancy and periconception. 12

In this category, it is clear that FA can be considered a protector for autism, especially when used in the preconception period and at the beginning of pregnancy, since research has shown that it reduces the risk of autism in the offspring. This beneficial effect cannot be confirmed if consumed during the rest of the course of pregnancy. It should be noted that many studies point to FA supplementation as having a beneficial effect in relation to ASD, since 8 of the 15 analyzed studies point to this effect.

### Increased Risk of ASD in Relation to Excessive Use of Folic Acid

Studies show that maternal FA supplementation, together with food fortification, has resulted in a population with superior parameters of AF than expected, and with an excess of nonmetabolized folate in the body. This may be associated with an increasing number of ASD cases. 13

### Inconclusive Association between Folic Acid Consumption and Autism Spectrum Disorder

It is still too early to reach a conclusion on the association between FA consumption and ASD. Studies show its protective effect against ASD, but others intrigue us with their investigations of alterations in the metabolic pathways of maternal folate, its potential role in DNA methylation through high maternal FA intake and its relationship with autistic traits. Finding the optimal level of maternal FA intake is difficult, but it may be the answer to these hypotheses. 14

The beneficial or harmful effect of FA on ASD cannot be stated. This requires intensified studies to discover the relationship of nutrition in different populations and the association with food fortification and additional FA supplementation. 15 16 The increase in the number of ASD cases may be linked to several factors, such as changing diagnostic criteria, increased information, and excess FA consumed by women of childbearing age. 17

Supplementation with periconceptional FA reduced neural tube defects by up to 70% and resulted in fortification of cereal products in several countries. The recommendation was that all women of childbearing age should consume 400µg/day of FA. It was also observed that the metabolism of maternal folate can vary between women, which may be involved with the increase in the number of cases of ASD.

Based on postpartum maternal blood samples, folate and vitamin B12 were analyzed in mothers who reported multivitamin supplement consumption at least in the 3 ^rd^ trimester of pregnancy. The diagnosis of ASD was observed through electronic medical records. The results can be understood as a “U” format, where the increased risk for ASD is at both extremes, when the supplement was consumed in excess and when it was consumed in small amounts. In contrast, moderate supplementation showed a protective effect in relation to ASD. 18

The maternal folate status may be related to ASD. Insufficient FA consumption may lead to hypomethylation of DNA, which is associated with neurodevelopment. It has also been found in some studies that FA consumption can be considered protective for autism.

The author indicates that repeated biological measurements of folate, vitamin B12 and homocysteine during the 1 ^st^ trimester of pregnancy are necessary, as well as genetic variants relevant to folate involved in carbon metabolism, and the epigenetic mechanisms. 19

The relationship of ASD with maternal use of micronutrients such as iron, zinc, vitamin D and AF were evaluated, and it was observed that the AF shows contradictory effects in relation to the TEA, which, in some studies, is presented as a protective effect and, in others, as causal effects. Thus, it was not possible to demonstrate whether or not there is a relationship between PA supplementation and ASD. Another finding in relation to ASD involves its relationship with glutathione, a product of the methionine cycle that depends on FA and B12, this element is involved in neuroprotection against oxidative stress and neuroinflammation in the brain. Research shows that glutathione is deficient in children with autism when compared with children with typical development. 20

## Conclusion

The use of FA in the preconception period and in the beginning of pregnancy is effective in preventing ASD. Therefore, early supplementation with FA is beneficial for neurodevelopment and, consequently, acts on the prevention of ASD. The present study deals strictly with the consumption of FA with the development of ASD, and the action of FA in the treatment of other neurological problems has not been reviewed. The study also did not aim to associate the use of FA with other drugs. The present study recommends that new investigations be performed to identify optimal doses and identify whether FA consumption from the 2 ^nd^ trimester can really be associated with the increase in the number of new cases of ASD as mentioned by some studies in the present review.
